# Supplementary material for: Stereotactic Body Radiation Therapy (SBRT) Plus Immune Checkpoint Inhibitors (ICI) in Hepatocellular Carcinoma and Cholangiocarcinoma
Source: Cancers (Basel). 2022 Dec 22;15(1):50. doi: 10.3390/cancers15010050 (PMC9817712; doi:10.3390/cancers15010050)
Supplement: Supplementary file 1 [file cancers-15-00050-s001.zip › cancers-2080554-supplementary.pdf]

Table S1: Immune-checkpoint Therapy Trials with LRT Eligibility

Trial Name	ICI Agent	Timing of LRT for Inclusion/Exclusion	Child-Pugh Classification
HIMALYA	Durvalumab +/- tremelimumab	Not specific	A
IMBrave 150	Atezolizumab	4 weeks	A
Check Mate 459	Nivolumab	4 weeks	A
CheckMate 40		Not specific	A and B7-8*
KEYNOTE 224	Pembrolizumab	Not specific	A
KEYNOTE 240		4 weeks	A

ICI-immune-checkpoint therapy LRT – locoregional therapy; *Child Pugh A5 or A6 for subjects in the nivolumab plus ipilimumab combination cohort and cabozantinib combination cohort; Child-Pugh B7 to B8 for subjects in the Child-Pugh B cohort
